# Social functioning in bipolar disorder: investigating the role played by comorbid physical illnesses and cognition

**DOI:** 10.1590/1980-5764-DN-2024-0188

**Published:** 2025-03-21

**Authors:** Isabela Martins Becattini Pereira, Lucas Machado Mantovani, Gabriel Anselmo Frota, Raphael Rocha Wenceslau, Juliana Cunha Matos, Breno Fiuza Cruz, Antônio Lúcio Teixeira, Izabela Guimarães Barbosa

**Affiliations:** 1 Universidade Federal de Minas Gerais, Instituto de Ciências Biológicas, Programa de Pós-Graduação em Neurociências, Belo Horizonte MG, Brazil.; 2 Universidade Federal de Minas Gerais, Faculdade de Medicina, Laboratório Interdisciplinar de Investigação Médica, Belo Horizonte MG, Brazil.; 3 Fundação Hospitalar do Estado de Minas Gerais, Instituto Raul Soares, Belo Horizonte MG, Brazil.; 4 Faculdade de Ciências Médicas de Minas Gerais, Belo Horizonte MG, Brazil.; 5 Universidade Federal de Minas Gerais, Escola de Veterinária, Departamento de Clínica e Cirurgia Veterinárias, Belo Horizonte MG, Brazil.; 6 Universidade Federal de Minas Gerais, Faculdade de Medicina, Departamento de Psiquiatria, Belo Horizonte MG, Brazil.; 7 The University of Texas Health Science Center at Houston, Department of Psychiatry and Behavioral Sciences, Texas, United States.; 8 Faculdade Santa Casa Belo Horizonte, Belo Horizonte MG, Brazil.

**Keywords:** Bipolar Disorder, Mania, Psychosocial Functioning, Functional Status, Social Interaction, Cognitive Dysfunction, Transtorno Bipolar, Mania, Funcionamento Psicossocial, Estado Funcional, Interação Social, Disfunção Cognitiva

## Abstract

Bipolar disorder (BD) is a psychiatric disorder associated with functioning and cognitive impairments, as well as a higher prevalence of physical disease comorbidities.

**Objective:**

To determine the main predictors of functioning in patients with BD.

**Methods:**

Thirty-five patients with BD type I in remission participated in this study. To better characterize the degree of impairment, 20 matched controls were also studied. Functioning was assessed through the Functioning Assessment Short Test (FAST) and the UCSD Performance-based Skills Assessment (UPSA), while cognition was assessed through the BAC-A. Current physical conditions were assessed and categorized according to the Cumulative Illness Rating Scale (CIRS). Regression analyses were performed to examine the relationship between functioning and clinical variables, global cognitive performance, and physical comorbidities in BD.

**Results:**

UPSA correlated positively with the BAC-A total score (r=0.488; p=0.025), years of education (rho=0.41; p<0.01), and CIRS total score (rho=0.394; p<0.001). CIRS was the only predictor that remained negatively and significantly correlated with the UPSA total score (R^2^=0.446, F (1, 33)=8.198, p=0.007).

**Conclusion:**

Patients with BD had poor functioning, with the primary determinant of poor functioning being the burden of physical illnesses. In addition, the low agreement between the FAST and UPSA scales suggests these tolls assess distinct constructs.

## RESUMO

O transtorno bipolar (TB) é um transtorno psiquiátrico associado a comprometimentos funcionais e cognitivos, além de maiores taxas de comorbidades físicas.

**Objetivo:**

O presente estudo tem como objetivo determinar os principais preditores de funcionamento em pacientes com TB.

**Métodos:**

Trinta e cinco pacientes com TB I em remissão participaram deste estudo. Para melhor caracterizar o grau de comprometimento, 20 controles também foram estudados. O funcionamento foi avaliado por meio do *Functioning Assessment Short Test* (FAST) e do UCSD *Performance-based Skills Assessment* (UPSA), enquanto a cognição foi avaliada por meio do Brief Assessment of Cognition in Affective Disorders (BAC-A). As condições físicas atuais foram avaliadas e categorizadas de acordo com a *Cumulative Illness Rating Scale* (CIRS). Análises de regressão foram realizadas para examinar a relação entre funcionamento e desempenho cognitivo global e comorbidades físicas no TB.

**Resultados:**

O UPSA correlacionou-se positivamente com a pontuação total do BAC-A (r=0,488; p=0,025), anos de educação (rho=0,41; p<0,01) e pontuação total do CIRS (rho=0,394; p<0,001). O CIRS foi o único preditor que permaneceu negativa e significativamente correlacionado com a pontuação total do UPSA (R^2^=0,446, F (1, 33)=8,198, p=0,007).

**Conclusão:**

Pacientes com TB apresentaram mau funcionamento, sendo o principal determinante de mau funcionamento a carga de doença física. Além disso, a baixa concordância entre as escalas FAST e UPSA sugere que essas ferramentas avaliam construtos distintos.

## INTRODUCTION

Functioning is a complex concept related to being able to perform social roles, such as working, studying, managing household chores, and maintaining personal relationships with others^1^. Functioning can also refer to the ability to perform activities of daily life in an autonomous and independent manner^2^. Aging and chronic physical illnesses are major drivers of functional impairment^3^. The presence of any physical comorbidity is an important factor related to disability, and specific combinations of chronic conditions are particularly relevant to increase functional impairment, such as diabetes and cardiovascular diseases^4,5^.

Bipolar disorder (BD) is a complex and chronic psychiatric disorder marked by a pattern of chronic and/or recurrent mood swings. Even with adequate mood stabilization and remission of mood symptoms, BD is associated with significant functioning impairment. Factors such as older age, low income, subsyndromal depressive symptoms, and poor executive functions, verbal memory, working memory, and verbal fluency, have been associated with functional impairment in BD^6,7,8^. The role of cognitive performance in functioning cannot be overlooked. Poorer global functioning in BD has consistently been associated with cognitive performance^9^. Furthermore, the occurrence of mood episodes can add significant functional burden to patients. While depressive symptoms can impact mainly work, autonomy, and interpersonal relationships, manic/hypomanic symptoms influence autonomy and financial skills^10,11^.

No previous study has simultaneously evaluated functioning in patients with BD considering both physical comorbidities and cognitive parameters. To control for the effect of mood episodes, we assessed patients with BD in euthymia. We also assessed the potential overlap of two functioning tools: one based on patients’ beliefs/opinion (Functioning Assessment Short Test – FAST) and the other based on structured clinical assessment (Performance-based Skills Assessment – UPSA). We hypothesized that poor cognition and the severity of chronic physical conditions are the main determinants of poor functioning in patients with BD.

## METHODS

### Subjects

This is a convenience sample study that recruited 35 patients with BD type I in remission and 20 matched controls. Participants were aged between 18 and 65 years old. Clinical assessment of research participants included demographic and clinical information, such as sex, age, length of illness. Current chronic physical conditions were assessed and categorized according to the Cumulative Illness Rating Scale (CIRS)^12^.

Patients were consecutively recruited from an outpatient psychiatric clinic specialized in BD at Hospital das Clínicas de Minas Gerais (EBSERH-HC/UFMG), Belo Horizonte. Patients were assessed with the Hamilton Depression Rating Scale (HDRS) (17-item version)^13^, and the Young Mania Rating Scale (YMRS)^14^ to determine the severity of depressive and manic symptoms, respectively. Inclusion criterion for BD was remission, defined by YMRS and HDRS scores lower than 7 points for 8 consecutive weeks. The only exclusion criterion for participants was illiteracy and participants whose Mini-Mental State Examination (MMSE) scores were below cut-off points indicative of cognitive impairment (see below). Controls were recruited from the community and had no history of psychiatric disorders or family history of such conditions.

All volunteers provided their written consent after a complete explanation of the procedures involved in the research protocol. Data were collected in 2021. The local institutional review board approved the study, which is in accordance with the 1975 Helsinki Declaration. All tests were administered by the same researcher (psychiatrist).

### Functioning assessment

Functioning assessment included the FAST^15,16^ and the UPSA^17^. The FAST is a brief questionnaire designed to assess the main functional difficulties in patients with BD. It has been translated and validated for use in Brazil^15^. It consists of 24 items covering six distinct functional areas:

- Autonomy;
- Occupational functioning;
- Cognitive functioning;
- Financial issues;
- Interpersonal relationships; and
- Leisure time.

The scale ranges from 0 to 72 points, with higher scores indicating worse functioning^16^. FAST scores between 0-11 indicate no functional impairment, between 12-20 indicate mild functional impairment, between 21 to 40 indicate moderate functional impairment, and above 40 indicates severe functional impairment^17^.

The UPSA^18^ is a test developed for the assessment of functioning using controlled scenarios that replicate daily life situations. It assesses basic skills across five areas:

- Understanding and planning;
- Finances;
- Communication;
- Mobility;
- Housework.

It has been translated and validated for use in Brazil^19^. The score for each domain varies between 0 and 20, resulting in a final score between 0 and 100. The closer to 100, the lesser functioning impairment.

### Cognitive assessment

The cognitive examination included the MMSE^20^ and the Brief Assessment of Cognition in Affective Disorders (BAC-A)^21,22,23^. The BAC-A comprises eight tasks (affective interference, verbal memory, working memory, motor speed, semantic and letter fluency, executive functioning, attention and motor speed) informing about the supposedly most affected cognitive domains in BD. It provides a global cognitive performance score.

We used the MMSE cut-off scores for cognitive impairment according to schooling years: 21 for illiterates, 22 for those with up to 5 years of education, 23 for those with 6 to 11 years of education, and 24 for those with 12 or more years of education^20^. Both psychiatric and cognitive assessments were performed on the same day, with each subject requiring approximately 90 minutes to be completed.

### Statistical analysis

Differences in dichotomous variables (sex and presence of clinical comorbidities) were tested using the Chi-square test. For continuous variables, means, medians, standard deviations, and total range are presented. All variables were tested for normality of distribution by the Kolmogorov-Smirnov test. Non-parametric Mann-Whitney tests were used for group comparisons. Statistical analyses were conducted using SPSS 22.0 (SPSS Inc., Chicago, Illinois). All statistical tests were two-tailed, with a significance level of α=0.05.

The scores from BAC-A were standardized into z-scores (M±SD: 0±1)^21,22^. The total score of BAC-A was calculated by summing the standardized z-score across the eight cognitive domains (Global performance score).

The correlational analysis between the variables among patients with BD was performed using Spearman’s test. A multiple regression analysis was performed whereby the dependent variable was UPSA and the predictors were years of education, BAC-A, and CIRS (variables p<0.05 on univariate analyses) in patients with BD. A backward elimination procedure was applied and variables with the highest p value were progressively deleted from the model. The final model retained variables with a significance level ≤0.05.

To analyze the UPSA-1-BR ability to determine functioning of patients with BD, its scores were compared to the FAST cut-off point using the Receiver Operating Characteristic (ROC) curve. McNemar’s test was conducted to verify the degree of agreement between the scales. In addition, Kappa’s test was performed to verify whether the UPSA-1-BR alterations were consistent with those identified by FAST in patients with BD.

## RESULTS

### Demographic, clinical features, cognitive, and functioning assessment

As shown in Table 1, patients with BD and controls presented similar distribution for age, sex, years of education, and physical comorbidities. All participants were cisgender. All patients were medicated, 71.4% (25/35) were in use of lithium, 40.0% (14/35) were in use of anticonvulsants, 28.6% (10/35) were in use of atypical antipsychotics, and 11.4% (4/35) were in use of antidepressants. On the BAC-A, the standard deviation of the z-scores for the global performance score was 1.09. Patients with BD presented a higher CIRS total score compared to controls (p<0.001); 57.1% (20/35) patients with BD scored 2/4 at Endocrine-Metabolic CIRS subscale; 8.6 (3/35) patients with BD scored 3/4 at Endocrine-Metabolic CIRS subscale; 71.7% (25/35) patients with BD scored 3/4 at Psychiatric CIRS subscale, and 28.6% (10/35) patients with BD scored 4/4 at Psychiatric CIRS subscale.

**Table 1:** Sociodemographic and clinical features of patients with bipolar disorder in comparison with controls.

	Patients with BD (n=35)	Controls (n=20)	p-value
Age in years [P50 (P25-P75)]	51.0 (37.0–58.0)	40.0 (34.5–45.0)	0.69*
Sex, female [N (%)]	22 (62.9)	16 (80.0)	0.19^†^
Education in years [P50 (P25-P75)]	11 (11–15)	11 (9.5–12)	0.23*
Duration of disease in years [P50 (P25-P75)]	21 (10–30)	---	---
HAM-D [P50 (P25-P75)]	2 (2–2)	---	---
Young [P50 (P25-P75)]	0 (0–0)	---	---
Diabetes Mellitus [N (%)]	3 (8.6)	0	0.253^‡^
Arterial Hypertension [N (%)]	11 (31.4)	3 (15)	0.182^‡^
Dyslipidemia [N (%)]	12 (34.3)	3 (15)	0.122^‡^
Hypothyroidism [N (%)]	9 (25.7)	1 (5)	0.083^‡^
Overweight [N (%)]	10 (28.6)	---	---
Obesity [N (%)]	12 (34.3)	---	---
CIRS [P50 (P25-P75)]	7 (5–8)	0 (0–0)	<0.001*
FAST [P50 (P25-P75)]	19 (8.5–25.5)	---	---
Autonomy [P50 (P25-P75)]	4 (2–5.5)	---	---
Occupational functioning [P50 (P25-P75)]	2 (0–5)	---	---
Cognitive functioning [P50 (P25-P75)]	4 (1.5–6.5)	---	---
Financial issues [P50 (P25-P75)]	0 (0–1.5)	---	---
Interpersonal relationships [P50 (P25-P75)]	3 (1–7.5)	---	---
Leisure time [P50 (P25-P75)]	3 (1–4)	---	---
UPSA [P50 (P25-P75)]	78 (68–85)	18.5 (17–20)	0.02*
Understanding and planning [P50 (P25-P75)]	16 (15–18.5)	18 (16–18)	<0.001*
Finances [P50 (P25-P75)]	16 (12–17)	18 (13.7–18.75)	<0.001*
Communication [P50 (P25-P75)]	13 (9–14)	20 (17–20)	<0.001*
Mobility [P50 (P25-P75)]	17 (13.5–17)	20 (15–20)	0.61*
Housework [P50 (P25-P75)]	20 (15–20)	89 (83–93.75)	<0.001*

Abbreviations: BD, bipolar disorder; N, subjects; P, percentile; HAM-D, Hamilton Depression Scale; Young, Young Mania Scale; CIRS, Cumulative Illness Rating Scale; FAST, Functioning Assessment Short Test; UPSA, UCSD Performance-based Skills Assessment.

Notes: *Mann-Whitney’s test

^†^Chi-square test

^‡^Fisher’s exact test.

### Functioning assessment

According to the FAST scale, 74.3% of patients scored above 11 points (p<0.001), indicating functioning impairment. According to the UPSA scale, 43.0% of patients scored above 75 points. When compared to controls, patients with BD had poorer UPSA total scores (p<0.001), particularly in the domains of understanding and planning (p=0.02), financial skills (p<0.001), communication (p<0.001), and transport (p<0.001).

### Functioning determinants in patients with bipolar disorder

As demonstrated in the Table 2, UPSA correlated positively with the BAC-A total score (r=0.488; p=0.025), years of education (rho=0.41; p<0.01), and CIRS total score (r=0.394*; p<0.001). CIRS was the only predictor that remained negatively and significantly correlated with the UPSA total score (R2=0.446, F (1, 33) = 8.198, p=0.007). There was no correlation between age and UPSA or FAST scores.

**Table 2:** Function determinants in patients with bipolar disorder.

	Age	Years of education	Duration of disease	CIRS	UPSA	FAST
Age	---	- 0.059	0.668*	0.557*	- 0.282	0.001
Years of education	- 0.059	---	- 0.156	- 0.310	0.411^†^	- 0.292
Duration of disease	0.668*	- 0.156	---	0.419^†^	- 0.077	0.269
CIRS	0.557*	- 0.310	0.419^†^	---	0.394^†^	0.214
UPSA	- 0.282	0.411^†^	- 0.077	0.394^†^	---	- 0.120
FAST	0;001	- 0.292	0.269	0.214	- 0.120	---

Abbreviations: CIRS, Cumulative Illness Rating Scale; UPSA, UCSD Performance-based Skills Assessment; FAST, Functioning Assessment Short Test.

Notes: *p<0.01

^†^p<0.05.

### Contrasting different functioning scales in patients with bipolar disorder

Of the 23 patients that presented functioning impairment as measured by the FAST, seven (30.4%) did not show impairment according to the UPSA scores. Conversely, three of the 15 patients (20.0%) with impaired functioning measured by UPSA did not present impairment on the FAST. The Kappa value was 0.2, indicating low agreement between the FAST and UPSA scales.

## DISCUSSION

In this clinic-based study involving euthymic patients with BD, the primary determinant of poor functioning was the burden of chronic physical illness. The findings also demonstrated a low agreement between the FAST and UPSA scales, suggesting that these two scales assess distinct constructs and may be best applied together to provide a more comprehensive evaluation of functioning in BD.

Previous studies reported poor functioning in BD, primarily during mania episodes^24,25^ or depressive states^24,25,26^. To control for the effects of mood episodes, only research participants in euthymia were recruited for this study. Previous studies have shown that just one third of patients with BD in remission recover their previous level of functioning^7^. Accordingly, compared to healthy controls, our sample exhibited mild to moderate functional impairment, particularly at being independent, working, and performing cognitive tasks. A recent meta-analysis demonstrated that individuals at risk for BD also demonstrated poorer functioning than controls^27^. Altogether, these findings suggest that poor functioning might be a trait marker of BD.

This is the first study to assess the agreement between measures of functioning based on patients’ beliefs (*i.e.,* FAST scale) and functioning evaluated by an experienced clinician through controlled environment tests (*i.e.,* UPSA). A higher proportion of patients with BD appear to present poor functioning according to FAST compared to the UPSA. One hypothesis is that self-assessment has a negative impact on functioning evaluation, as patients with BD might lack full awareness of their own capabilities, leading to inaccurate self-estimations of performance. Conversely, UPSA provides measurements related to the ability to perform activities of daily life in an autonomous and independent manner^28^. It is measured through role-playing tasks in a controlled environment, being less susceptible to influences such as insight, personal values, or contextual variations^29^. These methodological differences likely account for the discrepancies observed in functioning assessments between UPSA and FAST in BD. Therefore, both scales should be applied to provide a more comprehensive assessment of functioning in patients with BD, a point that must be addressed in future studies.

In the current study, the primary determinant of poor functioning was the burden of physical illnesses. A recent cohort study including patients from seven countries found that lower community functioning in BD was associated with depressive symptoms, lower levels of education, a greater number of prior mood episodes, and the presence of a comorbid substance use disorder^30^. Besides these predictors, the assessment of chronic physical diseases must be considered, also informing about mortality, hospital readmission, and prolonged hospital stays^31^. Indeed, physical comorbidities influence the functioning and the quality of life, particularly in severe mental illness^32^. CIRS evaluates the cumulative burden impact of physical comorbidities on heath. Patients with BD tend to have more comorbidities than controls, regardless of the age group considered^33,34^. However, it remains to be determined whether improved treatment or management of physical comorbidities can affect functioning of individuals with BD.

Of note, endocrine and metabolic conditions had a significant impact on the CIRS total score, considering that 65.71% of patients with BD had moderate or severe issues in the Endocrine-Metabolic CIRS subscale. This is related to the high incidence of overweight and obesity within this demographic^35,36^. Our study also demonstrated that patients with BD presented poor functioning independently of cognitive impairment. In fact, functioning is a construct influenced by sociodemographic factors, years of study, and biological processes such as a mild chronic inflammatory profile^2^. While cognitive impairments in patients with BD appear to strongly influence social and occupational domains of functioning, their relationship to global functioning is less pronounced^37^.

Our study has several limitations. First, the cross-sectional design precludes any causality assumption. Second, the small sample size limits the generalization of the findings to the broader population of patients with BD. The patients studied had a long disease duration and were followed at a specialized service. Third, while patients did not fulfil criteria for major neurocognitive disorder, it is possible that subclinical and/or mild cognitive symptoms, not captured by the limited cognitive assessment performed, were present. Conversely, the inclusion of only euthymic patients strengthens the study, as functioning is known to be impaired during mood episodes^38^. All patients were medicated with mood stabilizers and/or antipsychotics, which can influence metabolic parameters. Interestingly, while medications are often associated with weight gain, historical observations by authors such as Kretschmer, even before the advent of psychopharmacology, associated a somatic typology (*i.e. habitus picnicus*, characterized by abdominal fat pattern) to an increased risk of developing manic-depressive psychosis^39^. More recent evidence confirms that BD is associated with increased weight, regardless of treatment with antipsychotics and mood stabilizers^40^. The researcher was not blinded to participant status, as patients were recruited from a mood disorder clinic and controls from the community. Future research must address these limitations by including larger samples, deeper cognitive phenotyping (*i.e*., assessing additional domains with multiple tools), and longitudinal design to explore changes over time.

In conclusion, our findings indicate that patients with BD present impaired functioning, especially those with chronic physical comorbidities. A comprehensive treatment plan addressing both psychiatric and clinical/physical comorbidities might help prevent or mitigate trajectories of cognitive and functional impairment in BD.
